# Genomic analysis of LPS-stimulated myeloid cells identifies a common pro-inflammatory response but divergent IL-10 anti-inflammatory responses

**DOI:** 10.1038/srep09100

**Published:** 2015-03-13

**Authors:** Andrew Paul Hutchins, Yoshiko Takahashi, Diego Miranda-Saavedra

**Affiliations:** 1 Key Laboratory of Regenerative Biology and Guangdong Provincial Key Laboratory of Stem Cell and Regenerative Medicine, South China Institute for Stem Cell Biology and Regenerative Medicine, Guangzhou Institutes of Biomedicine and Health, Chinese Academy of Sciences, Guangzhou, Guangdong 510530, China; 2 World Premier International (WPI) Immunology Frontier Research Center (IFReC), Osaka University, 3-1 Yamadaoka, Suita, 565-0871 Osaka, Japan; 3 Center for iPS Cell Research and Application, Kyoto University, 53 Kawahara-cho, Shogoin Yoshida, Sakyo-ku, Kyoto 606-8507, Japan; 4 Centro de Biología Molecular Severo Ochoa, CSIC/Universidad Autónoma de Madrid, 28049 Madrid, Spain; 5 IE Business School, IE University, María de Molina 31 bis, 28006 Madrid, Spain

## Abstract

Inflammation is an essential physiological response to infection and injury that must be kept within strict bounds. The IL-10/STAT3 anti-inflammatory response (AIR) is indispensable for controlling the extent of inflammation, although the complete mechanisms downstream of STAT3 have not yet been elucidated. The AIR is widely known to extend to other myeloid cells, but it has best been characterized in macrophages. Here we set out to characterize the LPS-mediated pro-inflammatory response and the AIR across a range of myeloid cells. We found that whereas the LPS-induced pro-inflammatory response is broadly similar among macrophages, dendritic cells, neutrophils, mast cells and eosinophils, the AIR is drastically different across all myeloid cell types that respond to IL-10 (all bar eosinophils). We propose a model whereby the IL-10/STAT3 AIR works by selectively inhibiting specific pathways in distinct cell types: in macrophages the AIR most likely works through the inhibition of NF-κB target genes; in DCs and mast cells through indirect IRF disruption; and in neutrophils through IRF disruption and possibly also indirect NF-κB inhibition. In summary, no conserved IL-10/STAT3 AIR effectors were identified; instead a cell type-specific model of the AIR is proposed.

Inflammation is a crucial physiological response to infection and injury that must be rapidly and carefully managed to maintain the proper functioning of tissues with precise spatiotemporal control. Bacterial infection is a classic model of inflammation, where lipopolysaccharide (LPS, a major outer membrane component of Gram-negative bacteria) is an endotoxin that may eventually lead to sepsis, the uncontrolled release of pro-inflammatory cytokines^1^. Toll-like receptor 4 (TLR4) is a central mediator of the innate and adaptive immune responses to LPS and its activation ultimately results in cytokine production, among other cellular responses^2^.

Multiple pro- and anti-inflammatory molecules act to resolve and modulate the level of inflammation^3^,^4^, such as IL-10, a crucial negative regulator of inflammation. This potent anti-inflammatory cytokine^4^,^5^,^6^ was originally discovered as a critical factor produced by Th2 cells to suppress Th1 cell function^7^, but was later found to be produced by a wide-range of immune cells (e.g. macrophages, dendritic cells, T cells, B cells, mast cells and neutrophils) in response to inflammatory signals, and enacts a systemic anti-inflammatory response (AIR)^8^. The signaling pathways that culminate in the production of IL-10 are complex and might be cell type-specific and stimulus-dependent^8^,^9^.

The central role of IL-10 in deactivating immune cells in response to pathogenic invasion^10^,^11^ has been amply demonstrated by the numerous ways that pathogens have evolved to hijack the IL-10/STAT3 signaling pathway to prolong their survival. For example, *Leishmania major* and *Mycobacterium tuberculosis* both induce Il10 expression to activate an AIR through STAT3^12^,^13^. *Toxoplasma gondii*'s ROP16 kinase phosphorylates STAT3 in macrophages to activate the AIR, thereby escaping inflammation^14^,^15^. Lymphocytic choriomeningitis virus leads to increased levels of IL-10, poor clearance of the virus and defects in T cell responses^16^, and IL-10 plays a similar role in HIV infection^17^. Fascinatingly, cytomegalovirus and Epstein-Barr virus harbor IL-10 orthologues in their genomes^18^,^19^. All these observations suggest that high IL-10 levels are important in establishing persistent infections^11^. Besides its role in tempering excessive inflammation, IL-10 is essential for controlling the extent of inflammation in the intestine: the Il10 knockout mouse is the prototypical model of Crohn's disease^20^, and mutations in the human IL-10 receptor leads to severe inflammatory bowel disease with raised levels of TNFa^21^.

Although best studied in macrophages, the IL-10/STAT3 anti-inflammatory pathway has long been known to extend to other cells of the myeloid system^22^. For instance a macrophage and neutrophil-specific Stat3 knockout develops chronic enterocolitis^23^. In neutrophils, IL-10 has been implicated as a potent anti-inflammatory factor^24^ that can down-regulate ROS production^25^. IL-10 can also suppress TNFa and IL-6 production in rat mast cells^26^. In eosinophils, IL-10 treatment results in apoptosis and an AIR-like suppression of TNFa and IL-8^27^. However, a detailed molecular description of the mechanisms whereby IL-10 enacts the AIR in distinct myeloid cell types is currently lacking and is of fundamental importance for obtaining a global picture of how the various cells of the immune system combat infection in a dynamic manner^28^.

Here we have investigated the pro- and anti-inflammatory responses of five distinct myeloid cell types: macrophages, neutrophils, splenic dendritic cells (sDCs), mast cells and eosinophils. Of these, macrophages, neutrophils, sDCs and mast cells respond to IL-10 by phosphorylating STAT3 and suppressing pro-inflammatory cytokines. Using genomic technologies we systematically interrogated the transcriptional changes caused by LPS and IL-10. Macrophages, sDCs and neutrophils all respond very strongly to LPS, whilst mast cells and eosinophils showed a weaker response. We show that the LPS-induced pro-inflammatory transcriptional response is broadly similar among the myeloid cell types, particularly at the level of cytokine response. In contrast, the AIR is drastically different across the four myeloid cell types that respond to IL-10, suggesting that despite similarity in the myeloid cell phenotype and their response to LPS, their AIRs are cell type-specific. We further propose models of the AIR in the distinct cell types and suggest that in macrophages the AIR is primarily involved in the indirect inhibition of NF-κB signaling, whereas in neutrophils and sDCs the IL-10 response is mediated by IRFs. Our study brings a new global mechanistic insight into pro-inflammatory and anti-inflammatory responses in myeloid cells.

## Results

### The IL-10/STAT3-mediated AIR is activated in macrophages, neutrophils, sDCs and mast cells, but not in eosinophils

**Figure 1:**
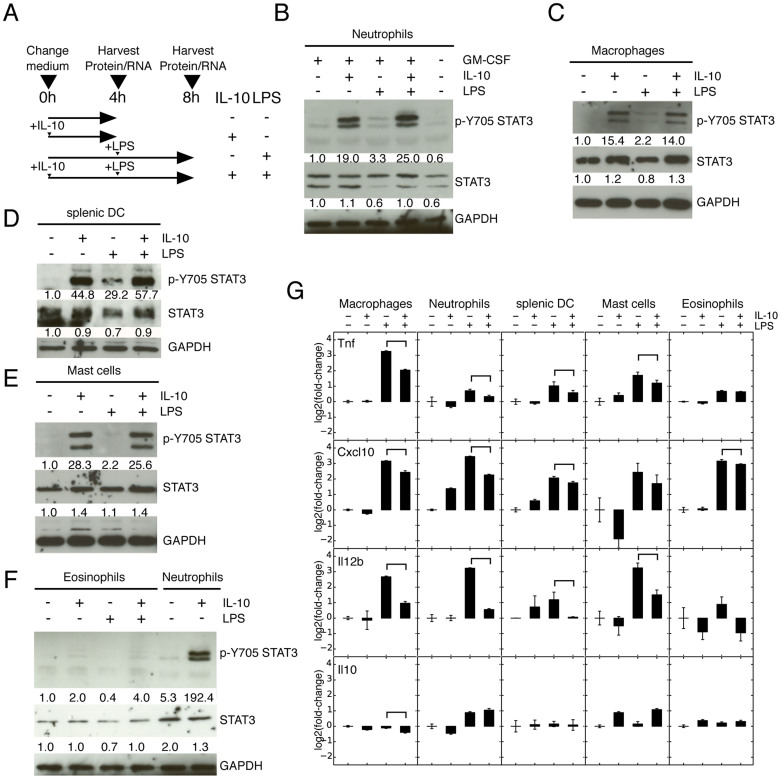
IL-10 leads to phosphorylation of STAT3 and activates an AIR in macrophages, neutrophils, sDC and mast cells, but not in eosinophils. (A) Schematic of treatment scheme used in this study. Macrophages, neutrophils, splenic sDC, mast cells and eosinophils were either purified (macrophages, neutrophils, sDC) or derived (mast cells, eosinophils) from mouse tissues, treated with IL-10 for 4 h and then subsequently treated with LPS for a further 4 hours. Upon addition of IL-10 STAT3 is phosphorylated in neutrophils (B), macrophages (C), sDCs (D) and mast cells (E), but not in eosinophils (F). Full-length blots are provided in Supplementary Figure 2. (G) qRT-PCR of the pro-inflammatory cytokines Tnf (TNFa), Cxcl10 (IP10) and Il12b, which are down-regulated when IL-10 is combined with LPS treatment except in eosinophils. Error bars are 95% confidence intervals, significantly down-regulated (p < 0.05) changes between +LPS and +IL-10+LPS are indicated. Genes must first be significantly up-regulated by LPS.

We explored whether the IL-10 AIR can be activated in five distinct myeloid cell types, including three primary cell populations (thioglycollate-elicited peritoneal macrophages, bone marrow (BM) neutrophils and sDCs) and two BM-derived cell types (mast cells and eosinophils). Cells were assessed for purity both by morphology (>80%) and flow cytometry (>90%) (Fig. S1 and see Materials and Methods). Additionally all cells were treated according to the same protocol (Fig. 1A), including pre-treatment with IL-10 for 4 h, followed by LPS stimulation for a further 4 h and harvesting. We decided to focus on these relatively short time periods so as to minimize secondary effects downstream of either IL-10 or LPS. We probed STAT3 for Y705 phosphorylation and observed robust phosphorylation in macrophages, neutrophils, sDCs and mast cells (Fig. 1B–E, S2), but in eosinophils only a very weak band was observed (Fig. 1F, S2). Murine eosinophils not showing phosphorylation of STAT3 is remarkable as human peripheral eosinophils have been shown to respond to IL-10 by initiating apoptosis and suppressing GM-CSF, TNFa and IL-8 cytokine production^27^, although in that case the IL-10 activity was not specifically linked with STAT3 activation. Potentially this could be a difference between human peripheral eosinophils and the mouse BM-derived eosinophils used here, or may be related to the long-term treatment of eosinophils with IL-10 and LPS as here we only treat the cells for 4 h. Neutrophils were treated overnight with GM-CSF as a maturation agent^29^ and most neutrophils adopted a mature phenotype and additionally showed IL-10 mediated suppression of Tnf (TNFa) (Fig. S3). Although GM-CSF has been reported to activate STAT3, at least transiently^29^, we could not detect Y705 STAT3 phosphorylation after overnight culture of neutrophils with GM-CSF (Fig. 1B, S2).

Next, to verify that the IL-10-mediated activation of STAT3 in fact executes an anti-inflammatory response, we quantified the expression changes of a set of inflammatory cytokines. Although the different cell types show different magnitudes of response to both LPS and IL-10 treatment, we can broadly say that macrophages, neutrophils, sDCs and mast cells present an AIR as determined by the suppression of Tnfa, Cxcl10 and Il12b upon IL-10 addition (Fig. 1G). As suggested by the lack of STAT3 phosphorylation in eosinophils, suppression of inflammatory cytokines was not observed (Fig. 1G). Potentially this is due to the low expression of IL-10 receptor genes Il10ra and IL01rb (Fig. S4A, S4B, S4C, S4D). The lack of STAT3 phosphorylation and corresponding lack of cytokine suppression indicates that eosinophils do not execute and anti-inflammatory response.

### Principal component analysis reveals cell type-specific expression profiles and suggests that the pro-inflammatory response has a cell type-independent component

**Figure 2:**
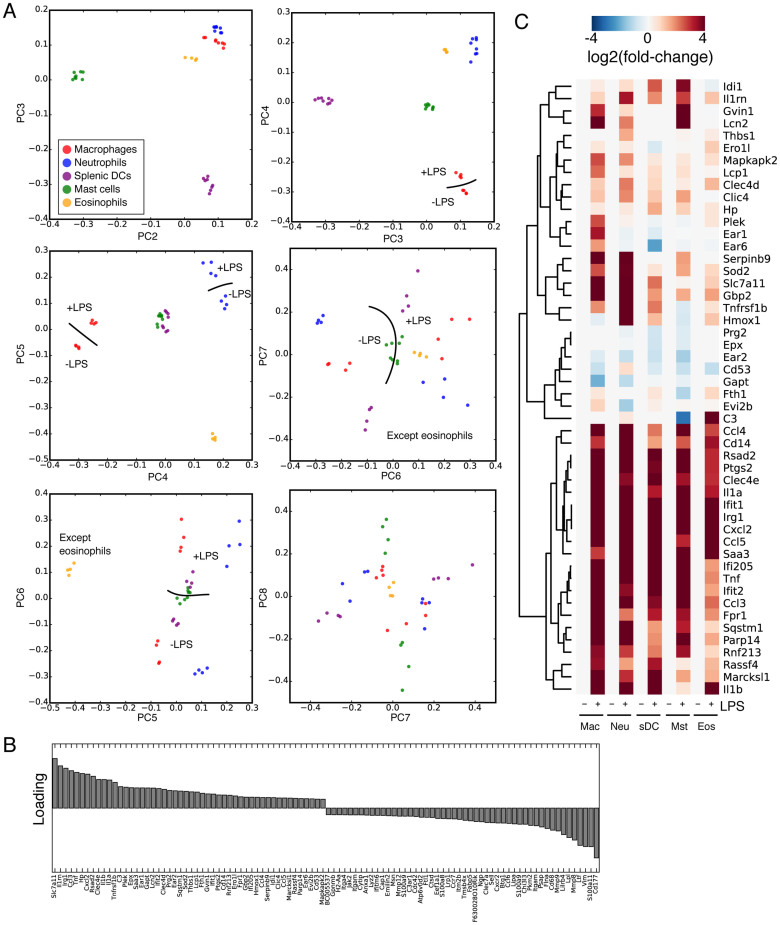
Principal componenet analysis of changes in myeloid gene expression. (A) Principal component (PC) analysis of the myeloid cells assayed in this study. PCs 2 through 7 are indicated, cell types are colored according to the key and where appropriate samples that segregate with and without LPS are indicated with a line showing the separation and ‘+LPS' and ‘-LPS' for the appropriate treatment. At no PC could we detect a gradient that corresponded to IL-10 treatment. (B) Loading for PC6, which correlates with LPS treatment. (C) Heatmap of the fold-change expression changes of the top 50 genes at the top of PC6.

RNA-seq experiments (Table S1) were performed to gain a broad picture of the transcriptional changes associated with the stimulation of all myeloid cell types with either LPS, IL-10, or both. Analysis of the biological replicates from independent mice reported Pearson correlations within the range of 0.7–0.9 (with most >0.8), indicating that our RNA-seq libraries are of high quality (Fig. S5). We performed principal component analysis (PCA) on all RNA-seq libraries to map the transcriptional variability of myeloid cells (both resting and stimulated). As expected, most of the variability arises from the identities of the various cell types rather than the treatments despite the potent effect that stimulation with LPS and IL-10 have on gene expression (Fig. 2A). PC2 through 5 mainly reflected cell type-specific gene expression programs, whilst a PC corresponding to LPS treatment did not appear until PC6 (Fig. 2A). At no point could we detect a PC that corresponded to IL-10 treatment. This indicated to us that the LPS response had some commonality between the cell types, but the IL-10 treatment conversely was cell type-specific. Along PC6 the gene loading indicated many pro-inflammatory factors in common between the cell responses (Fig. 2B), for example the cytokines Il1a, Il1b, Tnf (Fig. 2C).

To place our myeloid cell RNA-seq libraries into a broader context we re-analyzed publicly available RNA-seq datasets of related myeloid and lymphoid cells and clustered all samples by their pairwise coefficients of determination (R^2^). The macrophage and granulocyte cells cluster with previous RNA-seq samples, and the myeloid cells are all distinct from lymphoid cells types (Fig. S6).

### LPS-induced pro-inflammatory programs are relatively cell type-invariant

**Figure 3:**
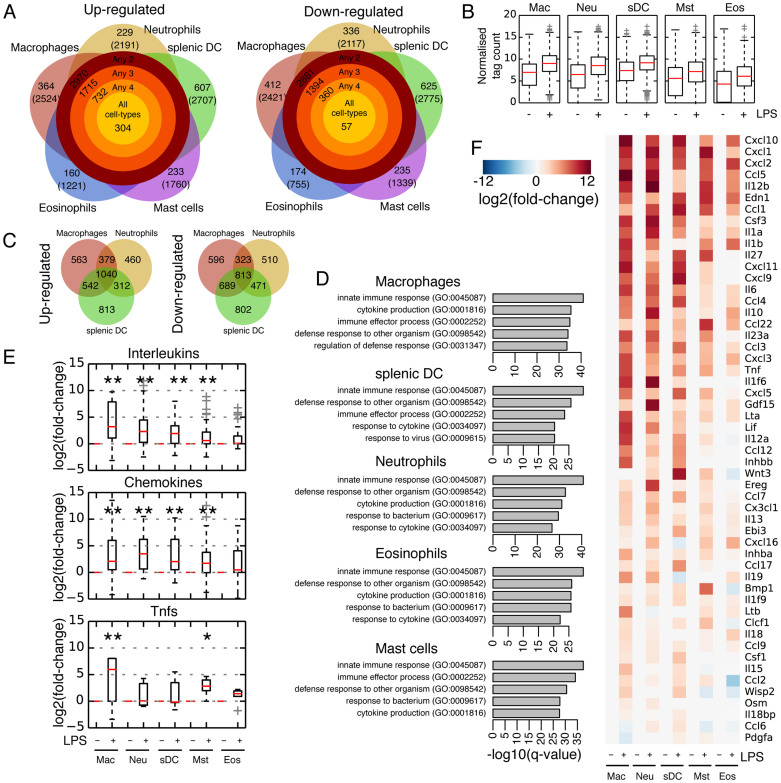
LPS endotoxin activates a potent, common pro-inflammatory response. (A) The number of transcripts up (left) and down (right)-regulated by LPS in each cell type and in different combinations of cells. Categories are exclusive, and the total number of genes regulated in the appropriate cell type is indicated in brackets. (B) Boxplots of relative levels of expression of LPS stimulated genes specifically up-regulated in the respective cell type. (C) Venn diagrams of up and down regulated transcripts in macrophages, neutrophils and splenic DCs. (D) Gene ontology analysis for up-regulated genes in the five cell types. (E) Boxplots for all expressed interleukins, chemokines and Tnf-family members. Mann-Whitney-U test: *p-value < 0.05, **p-value < 0.01. (F) Heatmap of the cytokines/chemokines up-regulated in at least 2 cell types, ordered by overall fold-change of expression upon addition of LPS.

When the distinct types of myeloid cell were treated with LPS they responded in different ways: whereas all five cell types upregulate ~1000–2000 transcripts (Fig. 3A; Table S2, S3), the overall magnitude of activation is much higher in macrophages, neutrophils and sDCs, whilst eosinophils and mast cells respond less strongly (Fig. 3B). If we focus only on the three strongest-responding cells (macrophages, neutrophils and sDCs), 2273 transcripts are upregulated in any 2 of the 3 cell types, indicating a substantial overlap in the LPS-mediated response, although some upregulated transcripts remain unique to each cell type (Fig. 3C). Functional (GO) analysis on the sets of upregulated transcripts indicates that all five cell types display a robust inflammatory response, as indicated by enriched GO terms for innate immunity and cytokine production suggesting a strong commonality in the LPS-mediated pro-inflammatory response (Fig. 3D).

In an alternate approach to the above thresholding strategy we also used weighted-gene correlation network analysis (WGCNA)^30^ to correlate genes changes with specific treatments (Fig. S7). WGCNA identified 1 module specific to LPS-treatment that was not also significant for any cell-type or IL-10 (Fig S7A). The module defined 649 genes, of which half were a subset of the genes identified by thresholding (Fig S7B, C). This alternate approach supports the idea that the LPS-response is relatively similar across the cell types, in agreement with the thresholding approach and the appearance of a principal component (PC6; Fig 2A) that corresponds to LPS stimulation.

We then analyzed the different families of signaling factors and looked at changes in gene expression. Cytokine, chemokine and growth factor gene annotations were manually collated from the gene ontology terms (e.g. GO:0005125 ‘cytokine activity') and divided into 20 ‘families' (interleukins, chemokines, growth factors etc), based on the annotation from the Cytokine Family Database. Their expression fold-changes upon LPS stimulation showed that the three families changing most significantly were interleukins, chemokines and TNF (tumor necrosis factor) cytokines (Fig. 3E and Fig. S8). Indeed, of the cytokines differentially regulated in at least 2 cell types almost all of them were either interleukins, chemokines or a TNF-family member, and the strongest responders by fold-change were consistently chemokines (Fig. 3F). These results suggest substantial similarity in the LPS response in each of the 5 cells, particularly at the level of cytokine/chemokine activation.

### IL-10/STAT3 suppresses distinct subsets of LPS-induced genes in distinct myeloid cell types

**Figure 4:**
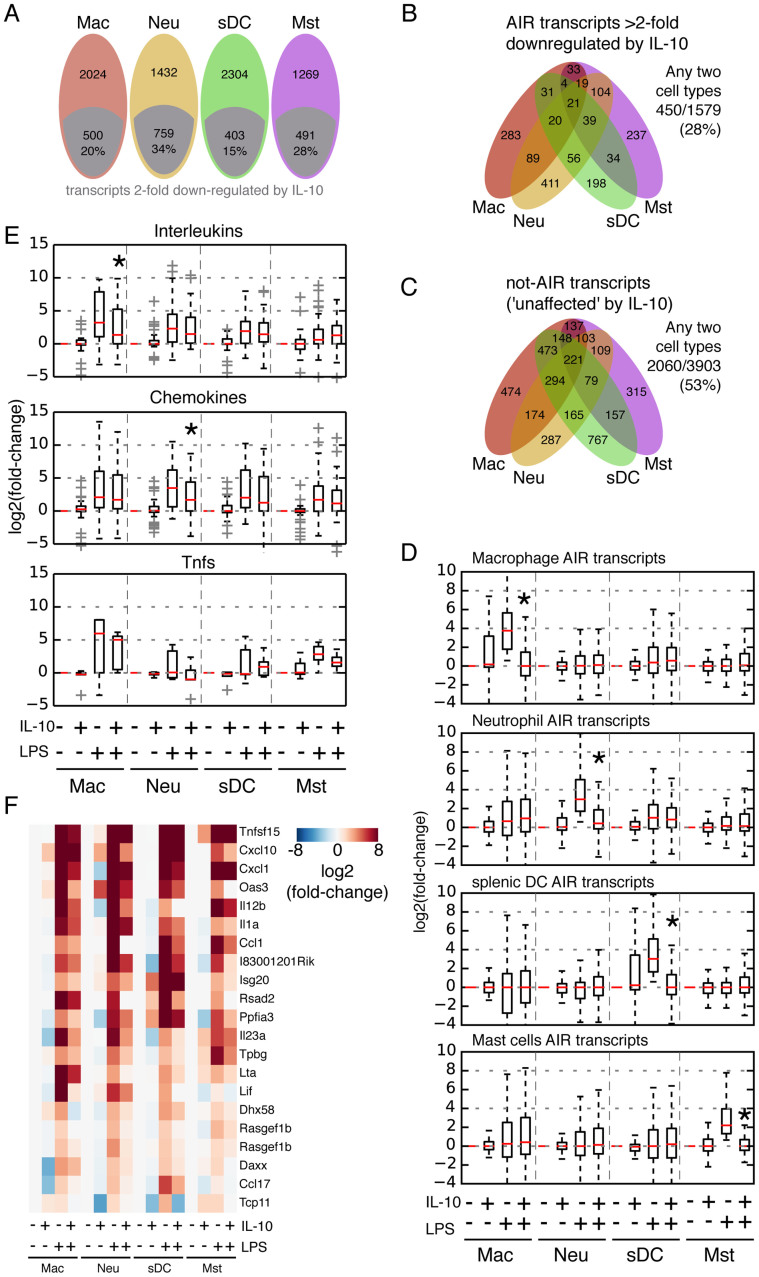
IL-10 suppression of the LPS-initiated pro-inflammatory response is divergent in the four myeloid cell types. (A) IL-10 suppressed transcripts (AIR transcripts) were defined as those transcripts declining at least 2 fold. (B) and (C) Transcripts were divided into two categories: (i) AIR transcripts – those transcripts that decline by at least 2 fold after LPS induction in at least one cell type and (ii) ‘not AIR' transcripts that did not decline at least 2-fold after IL-10 treatment. (B) Venn diagram of the IL-10 suppressed genes in the four strongest AIR-responding cell types, macrophages, neutrophils, sDCs and mast cells. (C) Venn diagram of ‘not-AIR' transcripts in the four strongest responding cell types. (D) Cell type-specific AIR transcripts are genuinely cell type-specific. AIR transcripts within the ‘any 2 cell types' category were removed from the analysis, but no other constraints were placed. Boxplot outliers are omitted for clarity (See also Fig S9). Mann-Whitney-U test: *p-value < 0.01 between +LPS and +IL-10/+LPS treatments. (E) Box plots showing the changes in gene expression caused by IL-10 on interleukins, chemokines and Tnf family members. Mann-Whitney-U test: *p-value < 0.05 between +LPS and +IL-10/+LPS treatments. (F) Heatmap of genes suppressed by IL-10 in at least three cell types.

In macrophages the IL-10/STAT3 AIR is known to suppress only a specific subset of all the genes induced by the LPS pro-inflammatory response^31^. Therefore the AIR does not have an indiscriminate effect on transcription, but specifically inhibits a number of pathways. Since neutrophils, mast cells and sDCs also respond to IL-10/STAT3, the anti-inflammatory response best described in macrophages may extend to other myeloid cells. To investigate this systematically, we divided the genes that were significantly upregulated by LPS into two classes: (i) ‘AIR genes', i.e. those that were downregulated at least 2-fold upon IL-10 stimulation; and (ii) ‘not-AIR genes', i.e. those whose expression did not fall by 2-fold. We found that only a subset of transcripts were downregulated by IL-10 in all myeloid cells investigated here, ranging from 403 in sDCs (15% of genes) to 759 in neutrophils (34% of genes) (Fig. 4A; Table S4). The number of AIR transcripts common to the four myeloid cell types was surprisingly low (n = 21), with just 450 AIR transcripts (28% of all AIR transcripts) down-regulated in any 2 cell types (Fig. 4B). Compare this to the not-AIR transcripts, which show a larger agreement, with 2060 (53%) genes common to any 2 cell types (Fig. 4C). This suggests that the AIR is fundamentally distinct across the four myeloid cell types. When we looked at the AIR genes from each cell type and their response in the other four cell types, not only are the genes very specific to each cell type (as expected since we removed the set of ‘any 2' genes from the analysis), but interestingly we also noticed that the AIR genes were only stimulated by LPS in their respective cell type (Fig. 4D; Fig. S9). This indicates that the cell type-specific component of the LPS pro-inflammatory response is also suppressed by IL-10 in a cell type-specific manner.

The expression levels of cytokines as an indication of the functional output confirmed that fundamental cell type-specific differences exist in the mechanism of action of the AIR: whereas interleukins are the major class of cytokines suppressed in macrophages, chemokines are especially suppressed in neutrophils (Fig. 4E). Looking at the set of AIR genes significantly downregulated in any 2 or any 3 cell types, the list is clearly dominated by cytokines and chemokines (Fig. 4F). Collectively our findings indicate that although macrophages, sDCs, neutrophils and mast cells can exert an AIR upon stimulation by IL-10, the underlying mechanisms are divergent across the distinct cell types.

### The IL-10/STAT3-mediated AIR employs various mechanisms to suppress the pro-inflammatory response

**Figure 5:**
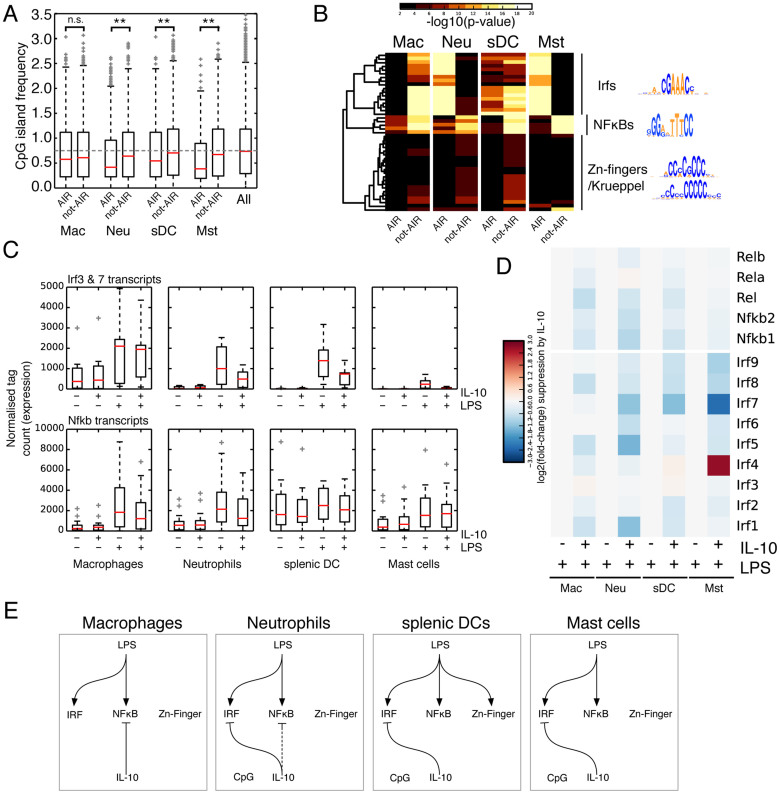
LPS and IL-10 employ different mechanisms in distinct myeloid cells. (A) CpG percent at the promoters of AIR and not-AIR genes. Promoter is defined as −450 bp +50 bp around the TSS. The numbers of CG dinucleotides were counted within that window and divided by the number of promoters in each cell type. The grey dotted line indicates a CpG island frequency of 0.75. Mann-Whitney-U test, **p-value < 0.01. n.s. not significant. (B) Motif enrichment at the promoters of AIR and not-AIR genes. (C) Changes in expression of Irf family transcripts and Nfkb family transcripts. (D) Boxplots of changes in Irf and Nfkb family transcripts. (E) A putative model for the differing mechanisms stimulated by LPS and IL-10 in the four cell types with both a pro and anti-inflammatory response.

To gain additional insights into the underlying mechanism initiating the pro-inflammatory response, we investigated the CpG content of the proximal promoters of AIR and not-AIR genes. Early LPS-responding genes are known to be CpG-rich, whilst later-responding genes are CpG-poor^32^. Additionally, chromatin remodeling at CpG-rich and CpG-poor promoters is known to be involved in the early and late stages of LPS-mediated gene activation^33^. Here we treated the cells with LPS for 4 h and thus expect the LPS-responding genes to be generally CpG-poor, which is the case for macrophages (Fig. 5A) and when treated with IL-10 the AIR genes show no significant difference in CpG frequency. Conversely neutrophils, sDCs and mast cells show a significant difference between AIR and not-AIR genes. Not-AIR genes show CpG levels similar to the rest of the transcriptome, but conversely AIR genes tend to be CpG poor. This suggests that in neutrophils, sDCs and mast cells one of the mechanisms of IL-10-mediated AIR involves CpG islands, which may reflect an alternate mode of chromatin remodeling^33^.

LPS stimulation is known to lead to the activation of several transcriptional pathways, particularly IRF3/7, NF-κB and AP-1 (Fos/Jun/etc)^34^. To gain some insight into the pathways being activated in the four cell types we took advantage of a property of TF binding, specifically that ChIP-seq experiments display a bias for TFs binding very close to the transcription start site (TSS) of regulated genes^35^. Using a collection of TF position weight matrices collated from the JASPAR, UniPROBE and HT-SELEX datasets^36^ we scanned the regions 450 bp upstream and 50 bp downstream from the TSS for over-represented motifs. We were surprised to observe a stark difference in the distribution of over-represented TF motifs in AIR and not-AIR genes (Fig. 5B). In all four cell types, IRF and NF-κB motifs were overrepresented in either AIR or not-AIR, whilst in sDCs (and less prominently in neutrophils) an as yet unidentified Zn-finger/Krueppel TF binding motif was detected among the not-AIR genes. We did not recover any AP-1 motifs, also thought to be involved in the TLR mediated transcriptional response^34^, likely because we analyzed near the TSS which is already enriched in AP-1 motifs, thereby highlighting one limitation of this approach. Critically, the over-represented motifs were unequally distributed amongst the AIR and not-AIR transcript sets (Fig. 5B; Fig. S10), suggesting that the AIR works primarily by inhibiting different TF pathways in different cell types: in macrophages the AIR most likely works through the inhibition of NF-κB target genes, whilst in sDCs and mast cells it appears to be through IRF disruption, and in neutrophils it is IRF inhibition and possibly also indirect NF-κB inhibition.

To explore this idea further, we looked at the down-regulation of IRF and NF-κB family members, reasoning that although alternate methods of suppression have been identified, particularly in macrophages, one important method is to modulate expression of the activating TF. In agreement with our model, in neutrophils Irf3, Irf7 and Nfkb family member transcripts are downregulated by IL-10, whilst in macrophages IL-10 does not affect the expression levels of Irf3 and Irf7 (Fig. 5C, 5D). In sDCs and mast cells Irf3 and Irf7 transcripts are down-regulated but NF-κB transcripts remain relatively unaffected (Fig. 5C, 5D). These observations lead us to propose the model shown in figure 5E, which summarizes the divergent AIR mechanisms across macrophages, neutrophils, sDCs and mast cells.

### IL-10/STAT3 is leading to activation of a diverse set of genes in myeloid cells

**Figure 6:**
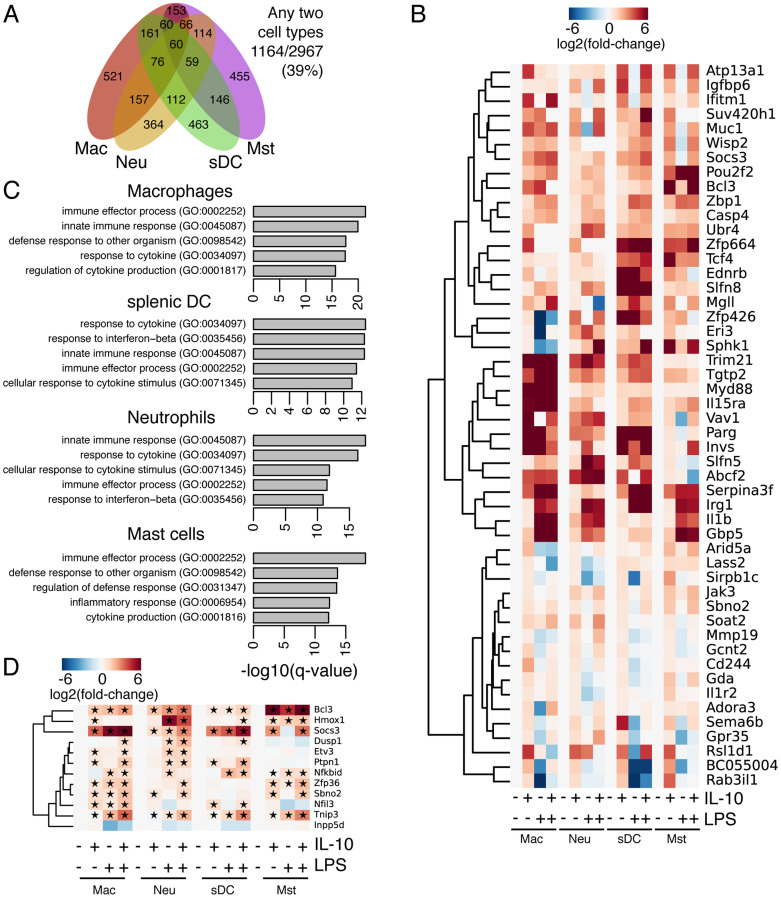
The IL-10/STAT3 target genes are highly divergent in macrophages, neutrophils, sDCs and mast cells. (A) Venn diagram overlap of genes significantly differentially regulated by IL-10. (B) Heatmap of the 50 genes (60 transcripts) induced by IL-10 in all four cell types. (C) Gene Ontology analysis of IL-10 activated genes in macrophages, neutrophils and sDCs. (D) Selected known IL-10/STAT3 target genes implicated in the AIR. The star indicates a fold-change of at least 1.5 fold in the respective treatment and cell type. Only one Sbno2 transcript is up-regulated whilst at the overall gene level its fold-change is only modestly up-regulated, hence it appears in panel B (transcript analysis).

We next looked at the transcripts that were significant and differentially upregulated by IL-10 after 4 h. The IL-10/STAT3 transcriptional program is divergent in the four cell types with a robust IL-10 response, showing just 50 genes in common among the three cell types (Fig. 6A; Table S5), but 39% of the IL-10 up-regulated genes are common amongst any two cell types, indicating at least some redundancy in IL-10 signaling, however WGCNA analysis failed to detect a common module for IL-10 signaling (Fig. S7), suggesting that the IL-10 signaling pathway is specific to the individual cell types. Of the 50 genes (60 transcripts) up-regulated in all four cell types (Fig. 6B), several of the genes we have previously identified as part of STAT3's cell type-independent function^35^,^37^, suggesting that the common part of the activation is cell type-independent and that it likely regulates general aspects of the JAK-STAT signaling pathway. Indeed, GO/KEGG analysis of the 60 common transcripts returns only a single significant category: ‘JAK-STAT signaling pathway' (KEGG:mmu04630, EASE score: 0.0971). Other analysis of gene ontology indicates many immune process pathways (Fig. 6C), but nothing specific for each cell type. Many of the genes are also stimulated by LPS alone (e.g. Fig. 6B), suggesting some degree of overlap between IL-10 and LPS signaling, something not unanticipated due to the close relationship between STAT3 and NF-κB in other biological contexts^38^. This nevertheless agrees with our model in Figure 5E: different pathways are being inhibited in the different types of myeloid cell, thereby underlining the diversity of the IL-10 response across myeloid cells.

Many known downstream targets of IL-10/STAT3 have been identified^6^,^39^. We noticed that of that list of known targets, only Bcl3, Socs3 and Sbno2 appear in the up-regulated list in response to IL-10 in all four cell types (Fig. 6B). Using a more relaxed cut-off of >1.5 fold induction we collected the known set of genes downstream of IL-10/STAT3 and marked which of the genes are up-regulated (Fig. 6D). The IL-10 activity on Nfkbid and Inpp5d are thought to be post-translational and we see no change in their expression; however all of the other targets, as expected, are up-regulated in macrophages (Fig. 6D). Interestingly, of the target genes only a small set are consistently up-regulated in all the cell types surveyed here, suggesting that known mechanisms of IL-10 action in macrophages may not immediately extend to other myeloid cell types.

We previously described a cell type-independent mode of STAT3 binding that leads to the activation of a set of genes across cell types as diverse as macrophages, embryonic stem cells, AtT-20 pituitary cells and CD4^+^ T cells, and whose role is to fine-tune the JAK-STAT pathway itself independently of the cellular context. When the new RNA-seq data described in this study was added we continued to see a consistent up-regulation of these genes (Fig. S11), thereby adding further support to our model for the cell type-independent binding mode of STAT3^35^.

## Discussion

IL-10 signals through STAT3 to activate a gene expression program and suppress pro-inflammatory genes^31^. It is also clear that STAT3 does not act to directly suppress pro-inflammatory genes, as new protein production is required for the AIR to occur^40^. Although the exact mechanism downstream of STAT3 has remained elusive despite extensive efforts^28^,^31^,^41^,^42^, in macrophages several genes act downstream of STAT3, particularly Bcl3, Tnip3, Etv3, Sbno2, Zfp36, Hmox1, Nfil3 and additionally the microRNA mir155^6^. These factors inhibit the pro-inflammatory response by a variety of mechanisms, some by targeting the inflammatory transcription factor NF-κB directly or by targeting specific mRNAs post-transcriptionally. Inpp5d (SHIP-1) has been reported as a target independent of STAT3 signaling^43^, although mir155 targets SHIP-1 mRNA for degradation and mir155 is suppressed by IL-10/STAT3^44^, suggesting a STAT3 link. However, no single factor has yet been able to explain all of the activities of IL-10 upon macrophages^4^.

Here we set out to determine whether a conserved AIR mechanism exists across cells of the myeloid system. Evidence for an AIR exists, especially in macrophages and neutrophils, although a full molecular characterization remains elusive. Here we show that four of the five myeloid cell types surveyed show evidence of an AIR initiated by IL-10. Macrophages, neutrophils, sDCs and mast cells all showed robust STAT3 phosphorylation and transcriptional suppression of cytokines. Curiously, eosinophils did not show strong phosphorylation of STAT3 nor suppression of inflammatory cytokines, suggesting a lack of an AIR. Eosinophils derived from human blood do show a response to IL-10, with increased apoptosis and suppression of TNFa and IL-18^27^. This discrepancy could be explained by the difference in species or cell origin (human peripheral eosinophils versus bone marrow-derived eosinophils), or could be due to some priming signal necessary for correct IL-10 activation of eosinophils that is missing in our system.

In agreement with results from macrophages^31^, we also observed that the IL-10 mediated AIR only suppresses a subset of genes stimulated by LPS. We can now extend that observation to other cells of the myeloid lineage, where IL-10 leads to the inhibition of between 10–33% of the LPS up-regulated genes. This observation indicates that whichever mechanism STAT3 uses to suppress pro-inflammatory genes, it is highly selective and does not inhibit gene expression globally. Instead the IL-10/STAT3 mechanism seems to inhibit different pathways in different cell types, mainly the inhibition of NF-κB target genes in macrophages, IRFs in sDCs and mast cells and both IRF and NF-κB target genes in neutrophils. We should highlight that these models remain hypothetical and do not immediately imply a direct action of IL-10 or STAT3 directly on these pathways. Complex interactions between signaling pathways are a common observation and in this system STAT3 and NF-κB are antagonistic, but in other systems STAT3 and NF-κB co-occur and even cause synergistic gene activation^38^.

The data set presented here is a fundamental resource for the exploration of IL-10 and the AIR in other myeloid cell types; the myeloid cells presented here respond to LPS in relatively similar ways, but show differences in their AIRs and especially in the genes responding to IL-10. Thus, the use of the cell type-specific gene expression signatures could be used to predict likely signaling events in systemic infection, where the exact activating cell may remain unclear, for example this technique was used to understand the cell types underlying the systemic Crohn's disease response^45^, which suggested that the response of granulocytes and dendritic cells is most important for this disease. As the transcriptional and epigenetic regulation of gene expression becomes better understood^46^ this data set can be used to model signaling events, including prediction of the complex transcriptional networks controlling the levels of inflammation. Similarly, as computational techniques become more advanced it may become possible to predict and even direct cell fate-determining processes and cell fate conversions. For example, the amalgamation of large amounts of gene expression data has led to a computational model to score and design strategies to engineer stem cell differentiation towards differentiated progeny^47^. Potentially these techniques could be applied to expanded immune system cells with much larger sets of perturbations to model behavior in response to infection and inflammation.

Our search for a unified mechanism of the IL-10/STAT3 mediated AIR in myeloid cells was hampered by the surprisingly low overlap between the IL-10 suppressed AIR genes across the various myeloid cell types^35^. Therefore, the identities of the majority of the effectors downstream of IL-10/STAT3 in the AIR remain unknown. From this and previous work we can summarize our knowledge about the pathways downstream of IL-10/STAT3: (i) The IL-10/STAT3 mediated AIR is common to multiple cells of the myeloid system; (ii) IL-10/STAT3 brings about the specific suppression of a subset of genes in multiple myeloid cells and not a general suppression; (iii) although all myeloid cells respond to IL-10, they do so by activating cell type-specific programs; (iv) it is likely that no single factor downstream of IL-10/STAT3 exists, instead several pathways may be in action. Analysis of the genome-wide binding patterns of STAT3 and the effectors of the LPS-mediated response may shed more light on the underlying mechanisms, and further work will be required to tease apart the exact combination of factors required for the AIR across the diversity of the immune cell repertoire.

## Methods

### Cell purification and cytokine treatment

Macrophages were extracted by peritoneal lavage as previously described^41^. Flow cytometry indicated macrophages were 83% Mac-1+ (Fig. S1A) before being allowed to attach to cell culture plates to select for adherent cells. Neutrophils were extracted by isolating BM from pooled mice (at least two mice in each experiment) and purified as described before^48^ albeit with some modifications: briefly, BM cells were first centrifuged through a 72/64/52% (v/v) Percoll (GE Healthcare) step gradient at 1500 g for 30 min. Next, neutrophils were collected from the bottom layer of cells and assessed for purity (routinely >80%) by May-Grunwald-Geimsa staining and 92% Ly6G/Gr-1 positive (Fig. S1B). Typical contaminants included lymphocytes with very few macrophage-like cells. Eosinophils were derived according to the protocol described by Dyer et al^49^: the cells were harvested and used for experiments on days 12, 14 and 16 and assessed by morphology and flow cytometry as 90% Siglec F^+^ (Fig. S1C). Mast cells were derived according to the protocol described by Jensen et al^50^: cells were assessed by morphology as >80% mast cells and were 98% FcREIa+ (Fig. S1D). Splenic DCs (sDCs) were purified from the spleen using the BD IMag ‘Mouse Dendritic Cell Enrichment Set –DM' according to the manufacturer's instructions. Purified sDCs were routinely >90% CD11c+ (Fig. S1E). GM-CSF (BioLegend) was used at a final concentration of 10 ng/ml, IL-10 (R&D Systems) was used at a concentration of 100 ng/ml and LPS (*E.coli* O55:B5; Sigma-Aldrich) was used at a concentration of 100 ng/ml. At the start of the assay and before treatment with IL-10 or LPS, the medium was replaced with fresh medium (RPMI1640 with 10% FCS).

### Western blots and qRT-PCR

Western blots were performed using typical laboratory procedures with antibodies to STAT3 (1:2000, C-20, Santa Cruz), phospho-Tyr705-STAT3 (1:1000, D3A7, #9145, Cell Signaling) and GAPDH (1:20000, AM4300, Ambion). qRT-PCR was performed on an ABI7900 using Realtime PCR and SYBR Green Realtime PCR master mix (TOYOBO). Primers used in this study: TnfF: 5′-TCCAGGCGGTGCCTATGT-3′, TnfR: 5′-CACCCCGAAGTTCAGTAGACAGA-3′, Cxcl10F: GACGGTCCGCTGCAACTG-3′, Cxcl10R: 5′-GCTTCCCTATGGCCCTCATT-3′, Il12bF: 5′-ATTGAACTGGCGTTGGAAGCAC-3′, Il12bR: 5′-TCTTGGGCGGGTCTGGTTTG-3′, Il10F: 5′-GATTTTAATAAGCTCCAAGACCAAGGT-3′, Il10R: 5′-CTTCTATGCAGTTGATGAAGATGTCAA-3′.

### RNA-seq and computational analysis

RNA from treated peritoneal macrophages, neutrophils, sDCs, eosinophils and mast cells was harvested with TRIzol (Life Technologies) according to the manufacturer's instructions. Biological replicates were generated from completely independent mice and sequenced on an Illumina HiSeq 2000. Sequencing and mapping statistics are detailed in table S1. RNA-seq data was analyzed essentially as described before^51^. Reads were aligned against ENSEMBL v67 (mm9) transcripts using RSEM (v1.2.1)^52^ and bowtie (v0.12.9)^53^. Raw tag counts were normalized for GC content using EDASeq (v1.8.0)^54^. Differential transcript expression was determined using DESeq (v1.14.0)^55^. Transcripts were considered as changing if they were significantly different (q-value < 0.1). Due to the conservative nature of DESeq and other differential expression algorithms, genes significant in one cell type were marked as differentially regulated in any other cell type if their fold-change was >1.5 fold, even if DESeq did not annotate them as significantly different. This allows a fairer comparison of similarities and differences between the various treatments. Weighted gene network correlation analysis was performed as described^30^. The raw sequence reads were deposited in GEO under the accession number GSE55385.

### Other bioinformatic analyses

The set of transcription factor (TF) genes was determined by amalgamating into a non-redundant set the predictions from the DNA-binding Domain database^56^ and AnimalTFDB^57^, plus those genes annotated with the Gene Ontology (GO) term GO:0005667 (‘transcription factor complex'). GO analysis was performed using GOSeq (v1.17.4)^58^, considering only GO terms containing between 20–500 genes. PSCAN^59^ was used for motif enrichment analysis using our own superlibrary of TF position weight matrices^36^. Other analyses were performed using glbase^60^.

## Supplementary Material

Supplementary Information Supplementary methods and figures

Supplementary Information Supplementary Dataset 1

Supplementary Information Supplementary Dataset 2

Supplementary Information Supplementary Dataset 4

Supplementary Information Supplementary Dataset 3

Supplementary Information Supplementary Dataset 5
