# Supplementary material for: Genomic analysis of LPS-stimulated myeloid cells identifies a common pro-inflammatory response but divergent IL-10 anti-inflammatory responses
Source: Sci Rep. 2015 Mar 13;5:9100. doi: 10.1038/srep09100 (PMC4650320; doi:10.1038/srep09100)
Supplement: Supplementary Information — Supplementary methods and figures [file srep09100-s1.pdf]

Genomic analysis of LPS-stimulated myeloid cells indicates a common pro-inflammatory response but divergent IL-10 anti-inflammatory responses

Andrew Paul Hutchins, Yoshiko Takahashi, Diego Miranda-Saavedra

List of Supplementary Files

Table S1. RNA-seq data from this study and library attributes and statistics.
Table S2. Lists of differentially expressed genes due to IL-10, LPS in each cell type.
Table S3. Lists LPS differentially genes specific to each cell type and in all 5 cell types, and any 4, 3 and 2 cell types.
Table S4. Lists AIR/not-AIR differentially expressed genes.
Table S5. Lists of IL-10 differentially regulated genes differentially genes specific to each cell type and in all 5 cell types, and any 4, 3 and 2 cell types.
Figure S1. Representative flow cytometry results for macrophages, neutrophils, dendritic cells, mast cells and eosinophils.
Figure S2. Full-length Western blots for STAT3 and pY705-STAT3 for Figure 1.
Figure S3. GM-CSF maturation of neutrophils enhances the anti-inflammatory response
Figure S4. RNA-seq expression of key IL-10 signaling pathway genes
Figure S5. Histogram of Pearson correlations for the biological replicates used in this study
Figure S6. Twenty families of signaling factors and their response to IL-10 and LPS
Figure S7. The full annotated version of Figure 5B
Figure S8. IL-10/STAT3 activates a 'core' of genes in a cell type independent manner

Supplementary Methods

Western blots, qPCR and flow cytometry

Western blots were performed using typical laboratory procedures with antibodies to STAT3 (1:2000, C-20, Santa Cruz), phospho-Tyr705-STAT3 (1:1000, D3A7, #9145, Cell Signaling) and GAPDH (1:20000, AM4300, Ambion). qPCR was performed on an ABI7900 using TOYOBO RT kits and SYBR. Primers used in this study: TnfF: 5'-TCCAGGCGGTGCCTATGT-3', TnfR: 5'-CACCCCGAAGTTCAGTAGACAGA-3', Cxcl10F: GACGGTCCGCTGCAACTG-3', Cxcl10R: 5'-GCTTCCCTATGGCCCTCATT-3', Il12bF: 5'-ATTGAACTGGCGTTGGAAGCAC-3', Il12bR: 5'-TCTTGGGCGGGTCTGGTTTG-3', Il10F: 5'-GATTTTAATAAGCTCCAAGACCAAGGT-3', Il10R: 5'-CTTCTATGCAGTTGATGAAGATGTCAA-3', Gata1F: 5'-ACTTTCCCAGTCCTTTCTTCTCTCC-3' Gata1R: 5'-GCCGTTGCTCCACAGTTCAC-3'. Antibodies used for flow cytometry: Mac-1-biotin (M1/70, BD Pharmingen), Ly6G/Gr-1-biotin (RB6-8C5, BD Pharmingen), Siglec-F-PE (E50-2440, BD Pharmingen), FcREIa-FITC (MAR-1, Biolegend),

anti-CD11c (N418, Biolegend) and Streptavidin-FITC (Biolegend). Flow Cytometry was performed on a BD Facs Calibur.

Figure S1

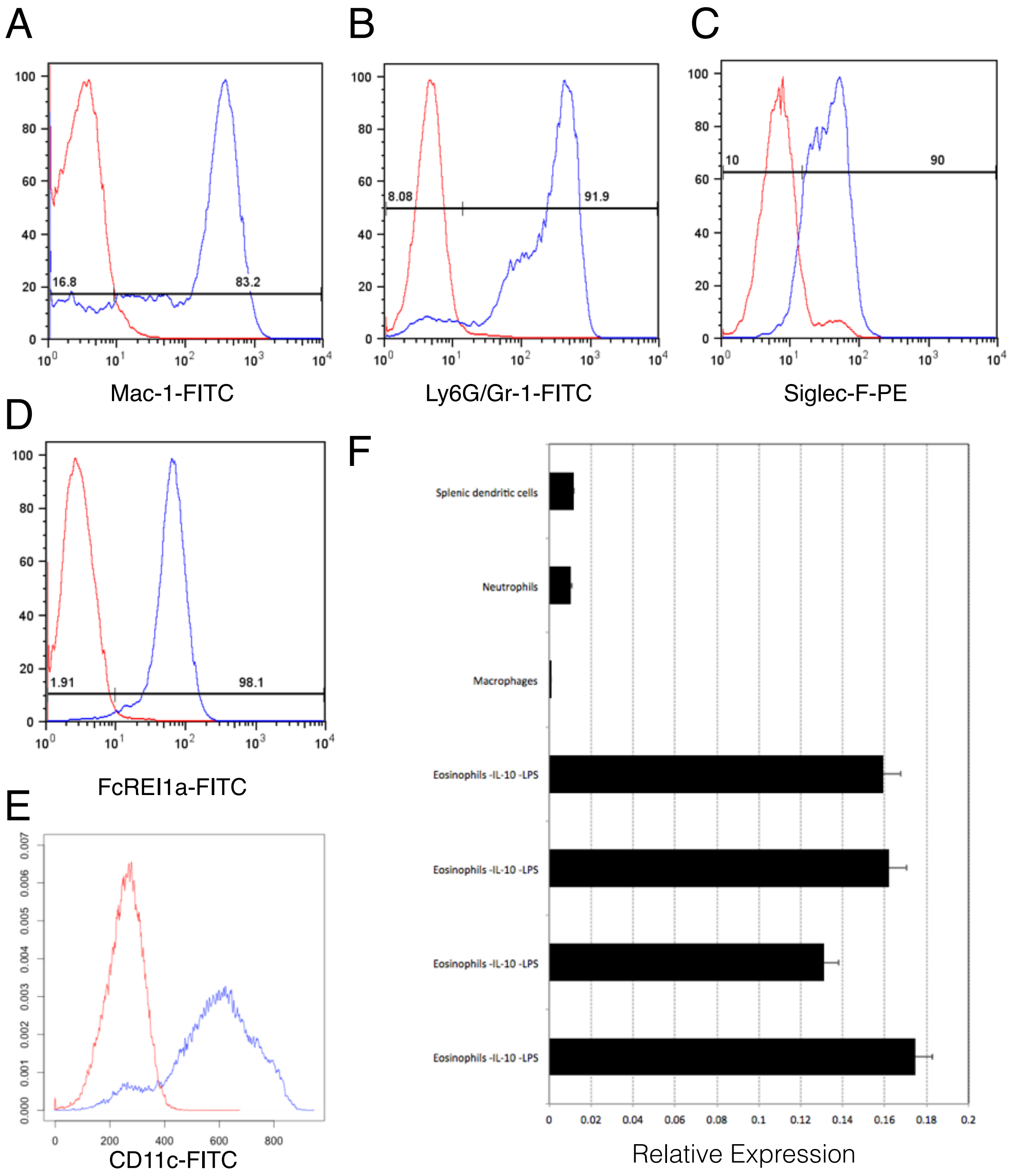

Figure S1. Representative flow cytometry results for (A) Macrophages stained with anti-Mac-1 (CD11b/Itgam), (B) Neutrophils stained with anti-Gr-1-FITC (C) Eosinophils stained with anti-Siglec-F-PE and (D) BMMC stained with anti-FcREIa-FITC. (E) splenic dendritic cells stained with anti-CD11c-FITC. Red histograms are unstained cells, blue histograms are stained with the appropriate antibody. (F) qRT-PCR measure of Gata1 expression in eosinophils. Expression is measured relative to Gapdh expression.

Figure S2

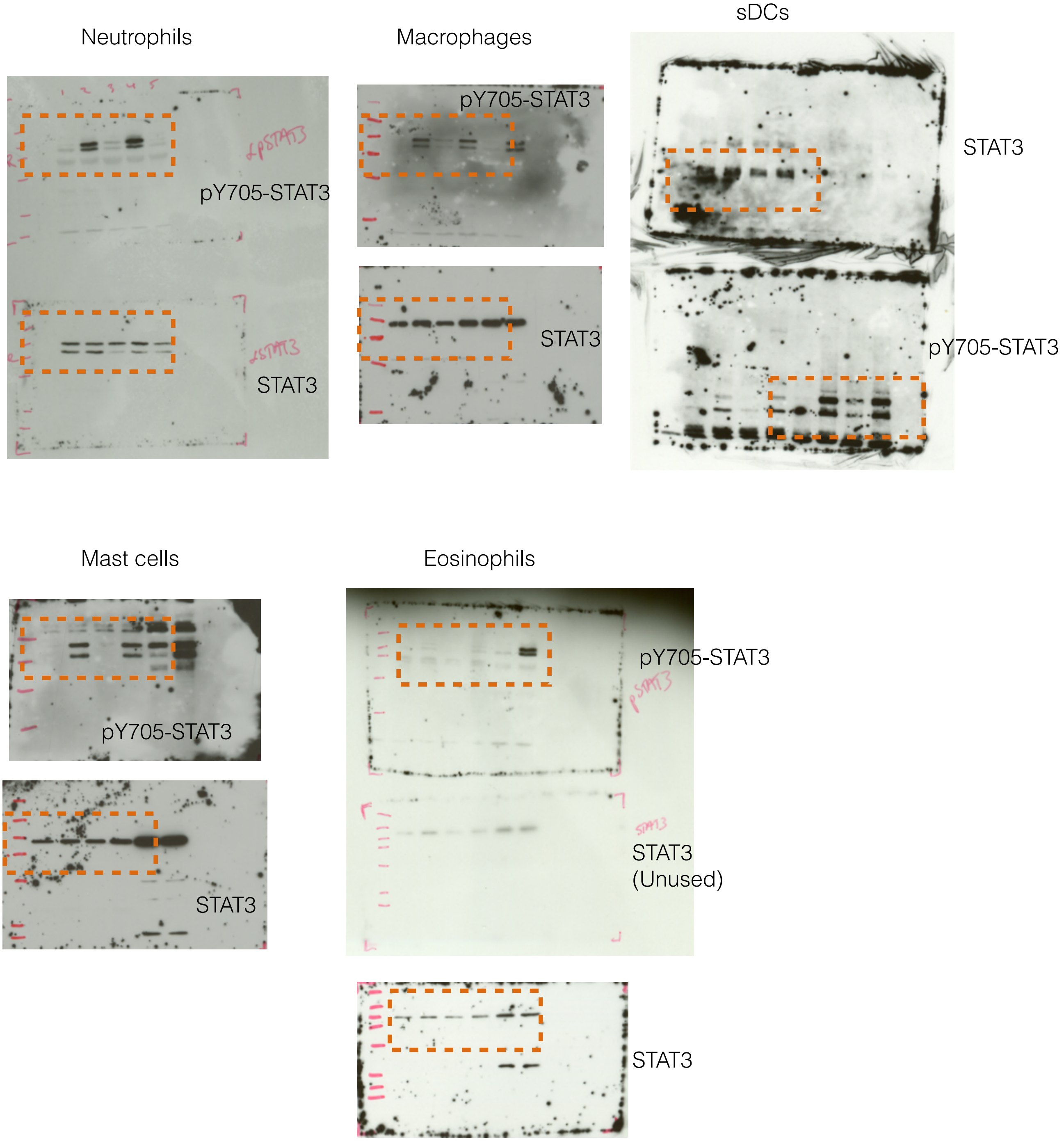

Figure S2. Full length Western blots for STAT3 and pY705-STAT3 for Figure 1. The approximate locations of the crop are marked with an orange box. Antibodies used were anti-STAT3 (1:2000, C-20, Santa Cruz) and phospho-Tyr705-STAT3 (1:1000, D3A7, #9145, Cell Signaling).

Figure S3

A

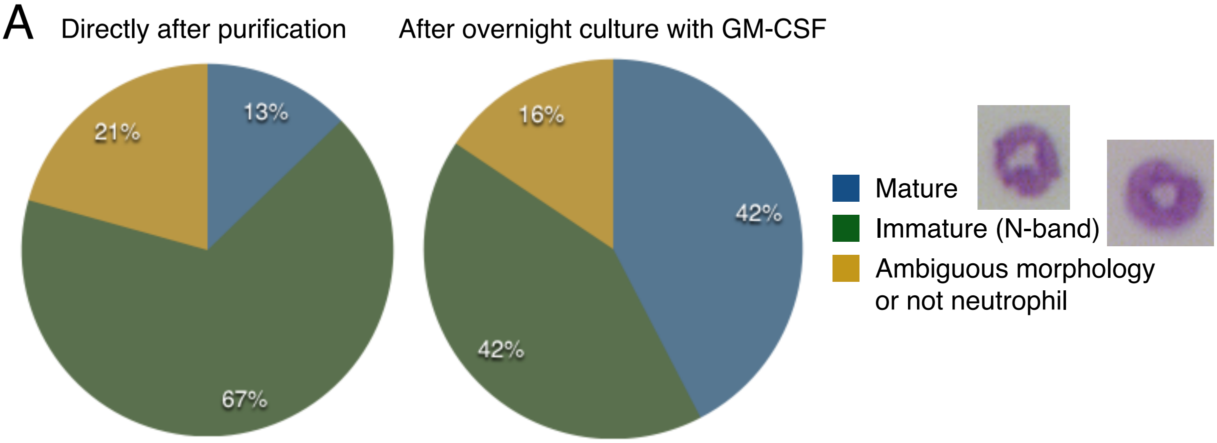

B

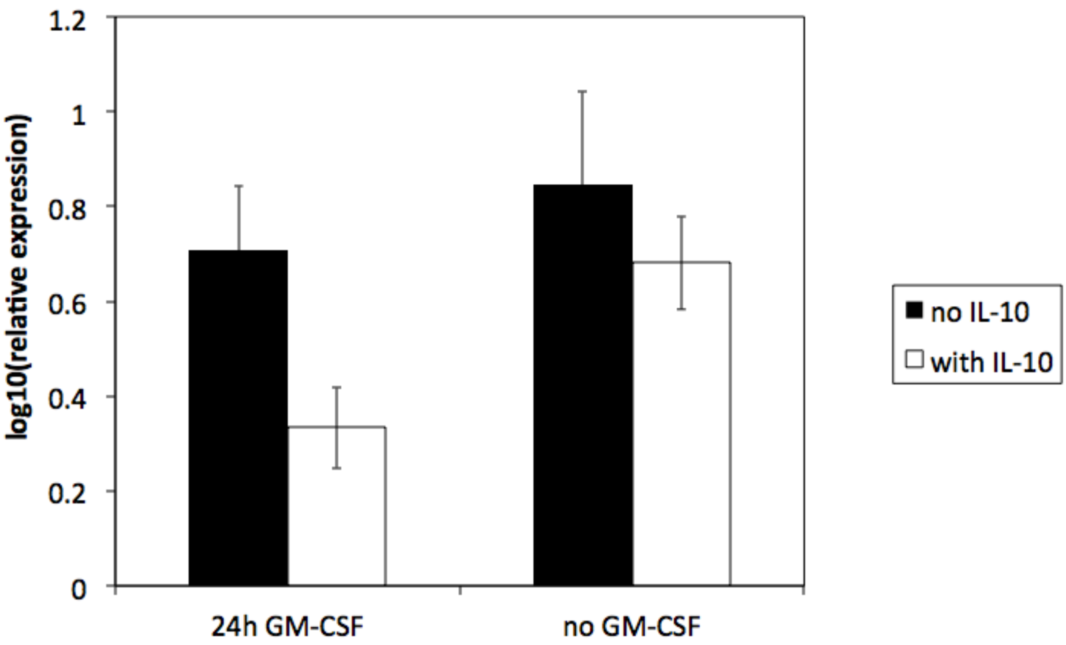

Figure S3. GM-CSF maturation of neutrophils. (A) Morphology of neutrophils immediately after purification from the bone marrow or after 24 hours of treatment with 10 ng/ml GM-CSF. (B) Suppression of Tnf (TNFa) mRNA is improved after maturation overnight.

Figure S4

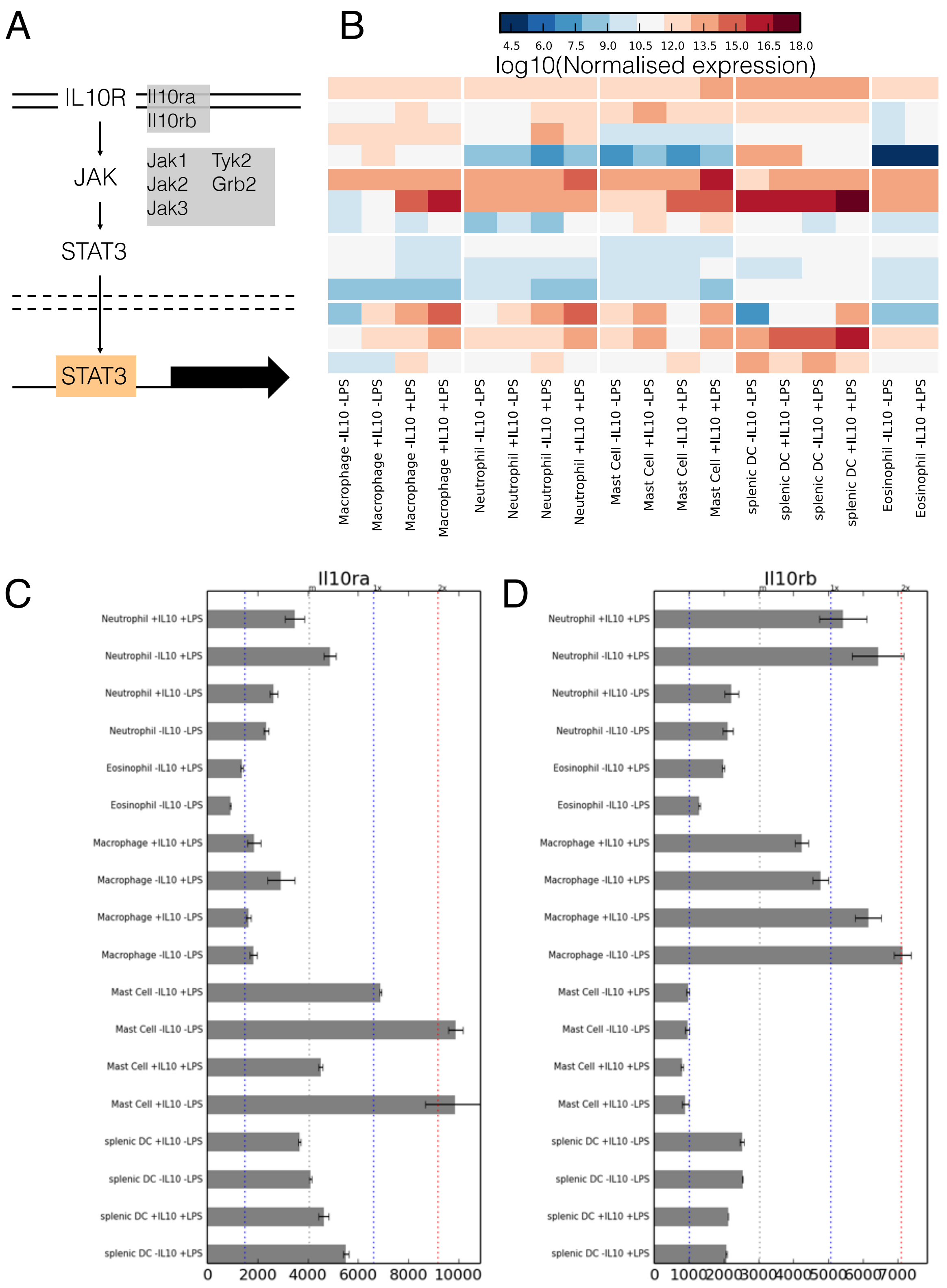

Figure S4. RNA-seq expression of key IL-10 signalling pathway genes. A. Schematic of IL-10/ STAT3 signalling pathway activation. Important genes are shown in grey boxes. B. Heatmap of RNA-seq gene expression for a selection of IL-10/STAT3 signalling related genes. C and D. Normalised expression tag counts for IL10ra (C) and Il10rb (D). Error bars are standard error of the mean between the biological replicates.

Figure S5

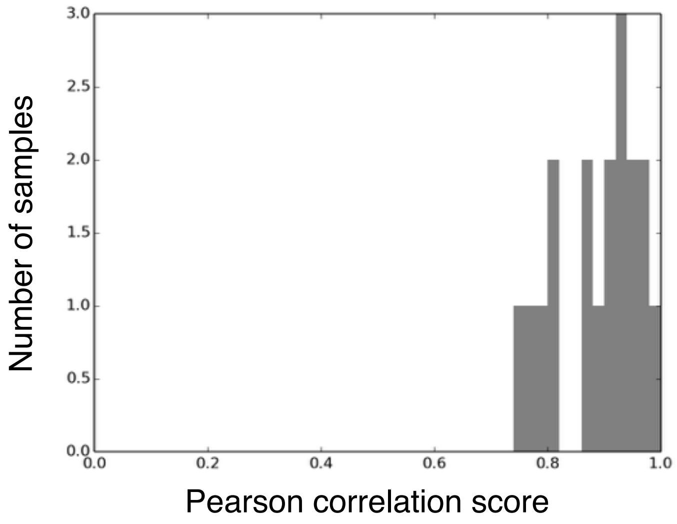

Figure S5. Histogram of Pearson correlation scores for biological replicate RNA-seq samples in this study.

Figure S6

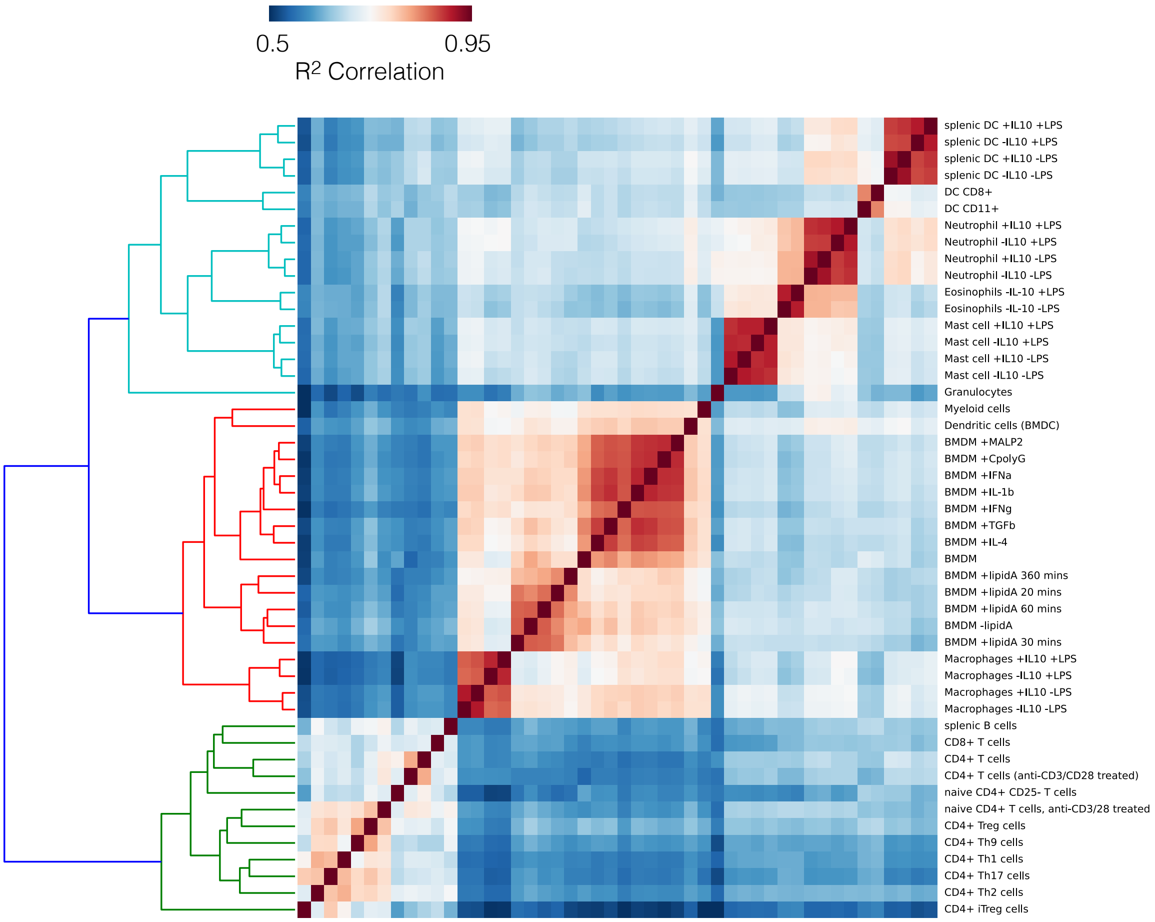

Figure S6. Global comparison of myeloid and lympohid cell type expression signatures. RNA-seq data from a selection of mouse immune cell types were hierarchically clustered based on their pair-wise coefficient of determination (R2). Macrophages cluster close to other myeloid cell types, but remain distinct from bone-marrow derived macrophages (BMDM) and dendritic cells. Granulocytes (neutrophils, eosinophils, mast cells) form their own branch, away from macrophage cells, but still within the myeloid cell branch. Lymphoid cells form a separate cluster distinct from other myeloid cells. Other RNA-seq libraries reanalyzed were GSE20898 (Wei et al., 2011, PMID:21867929), GSE28666 (Hebenstreit et al., 2011, PMID:21654674), GSE29209 (Grabherr et al., 2012, PMID:21572440), GSE31530 (Hutchins et al., 2012, PMID:22323479), GSE34550 (Hutchins et al., 2012, PMID:22884873), GSE36026, GSE38371 (Ostuni et al., 2013, PMID:23332752), GSE38892 (Bhatt et al., 2012, PMID:22817891), GSE39524, GSE39756 (Li et al., 2012, PMID: 22992523), GSE40350 (Ring et al., 2012, PMID:23104097), GSE40463 (Vahedi et al., 2012, PMID:23178119), GSE43504 (Barlow et al., 2013, PMID:23352430), GSE55385 (This study).

Figure S7

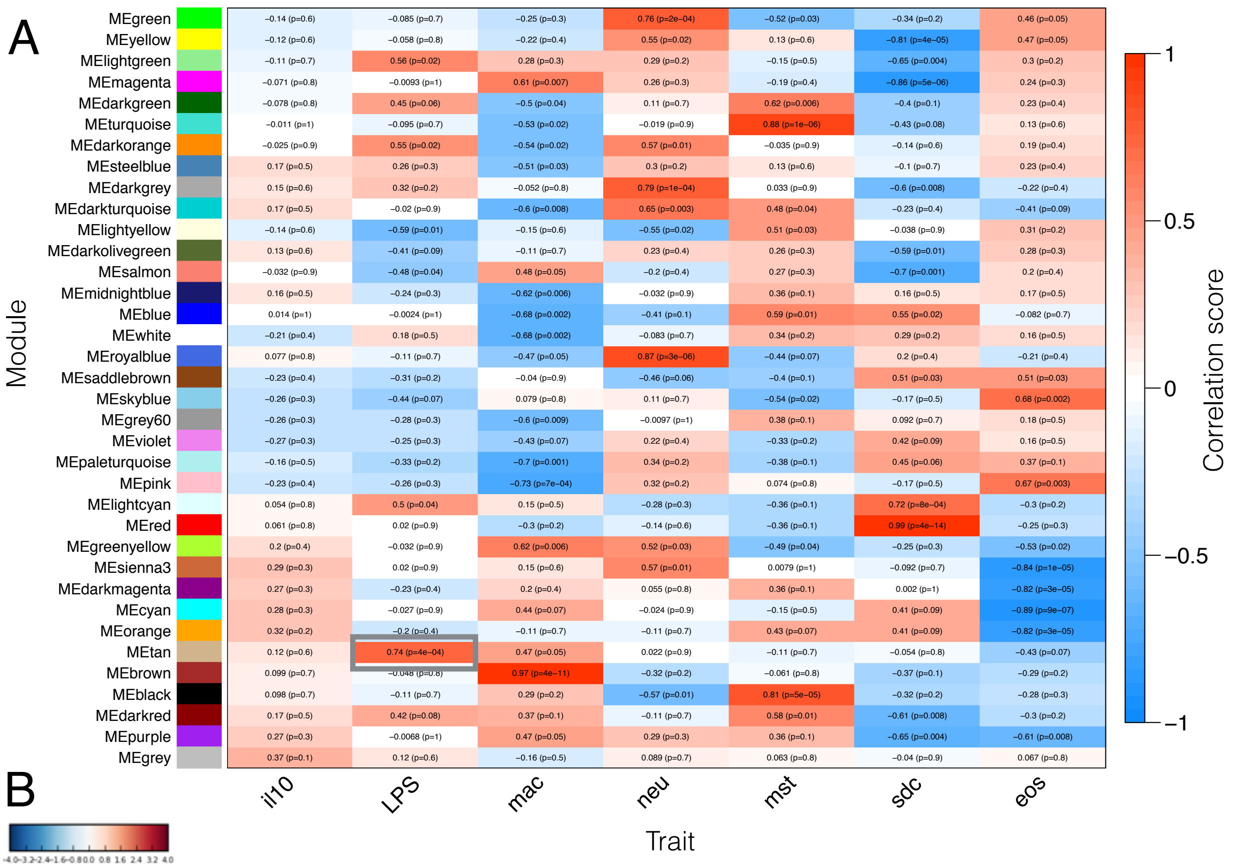

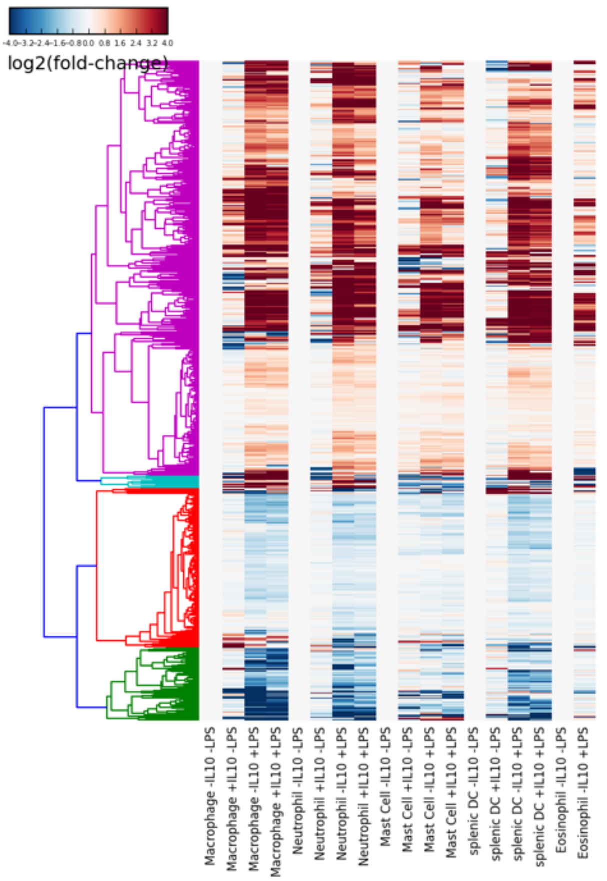

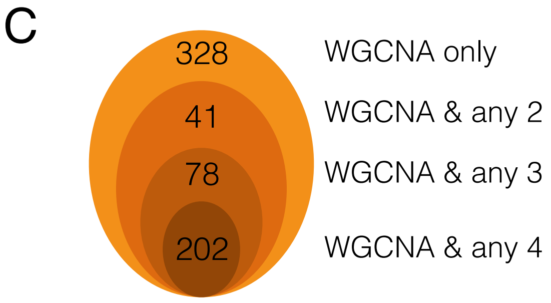

Figure S7. Weighted gene correlation network analysis (WGCNA) reveals a conserved regulatory module specific to LPS treatment, but could not recover and IL-10 specific module. Suggesting the LPS-response is relatively cell type-invariant, but the IL-10 and AIR is cell type-specific. (A) labelled heatmap of WGCNA detected modules, their correlation score and p-value (in brackets). (B) Genes belonging to the 'tan' module, associated with LPS stimulation. (C) Of the 649 genes in the LPS-specific ('tan') module 334 are in common with the genes detected by threshold analysis.

Figure S8

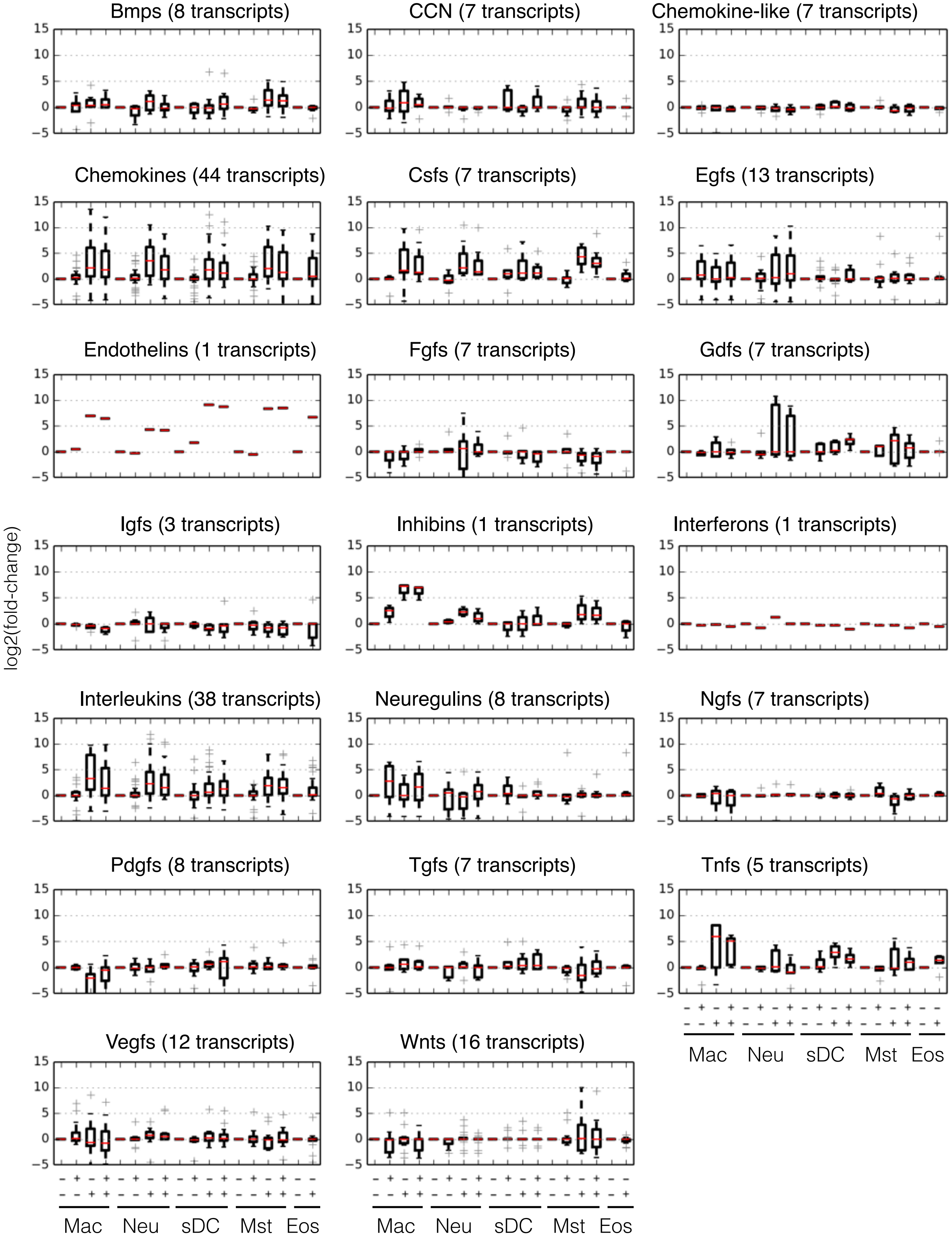

Figure S8. Twenty families of signalling factors and their response to IL-10 and LPS. The families are indicated and the number of expressed transcripts in our RNA-seq data is indicated. Expression is log2(fold-change).

Figure S9

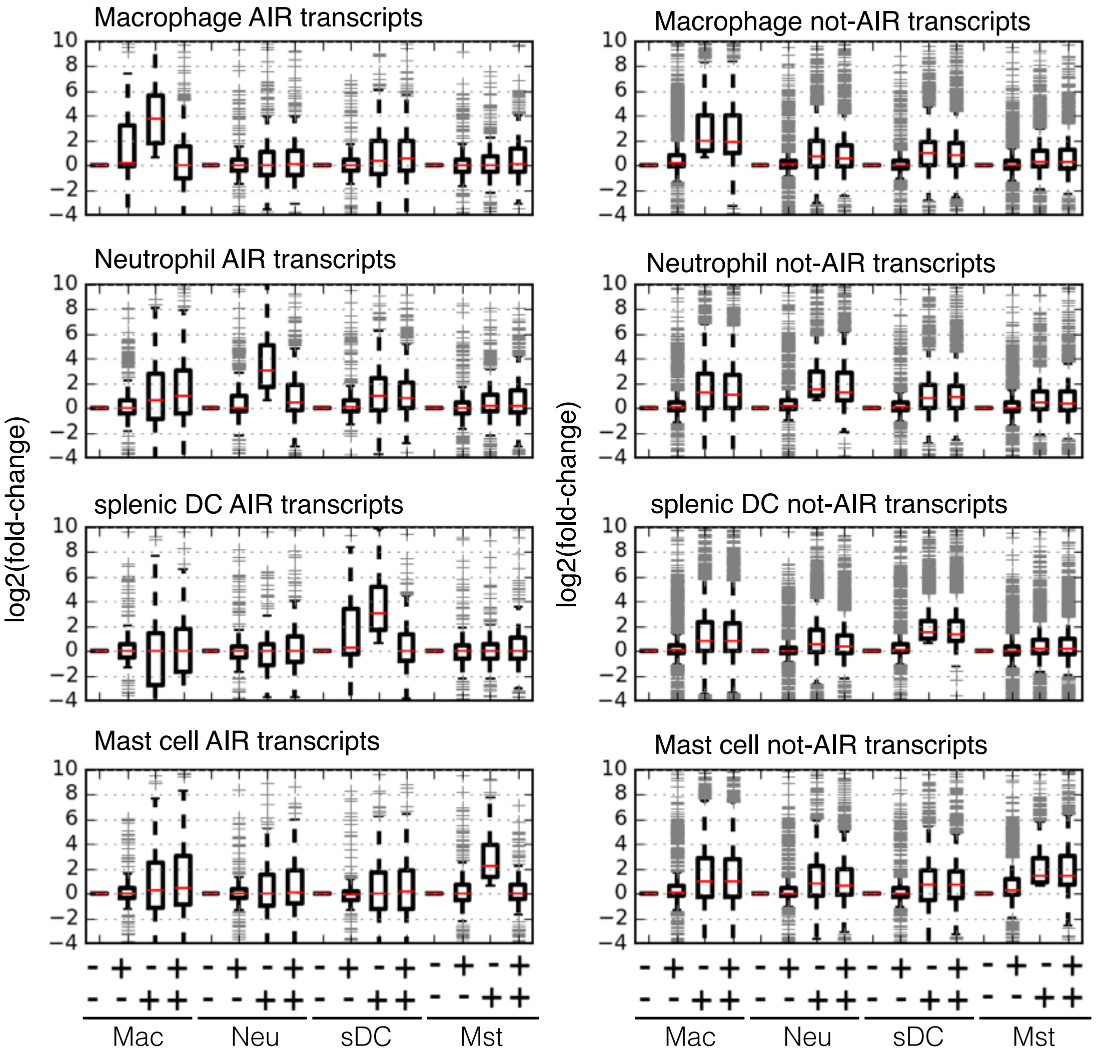

Figure S9. AIR transcripts are cell type-specific. In each case only the AIR genes specific to the indicated cell type are significantly down-regulated when IL-10 is added. Transcripts that were in 'any 2' cell types (The Venn overlap from Fig. 4B) were removed to give only cell type-specific genes and then compared in the other cell types. AIR genes are indicated on the left, not-AIR genes on the right.

Figure S10

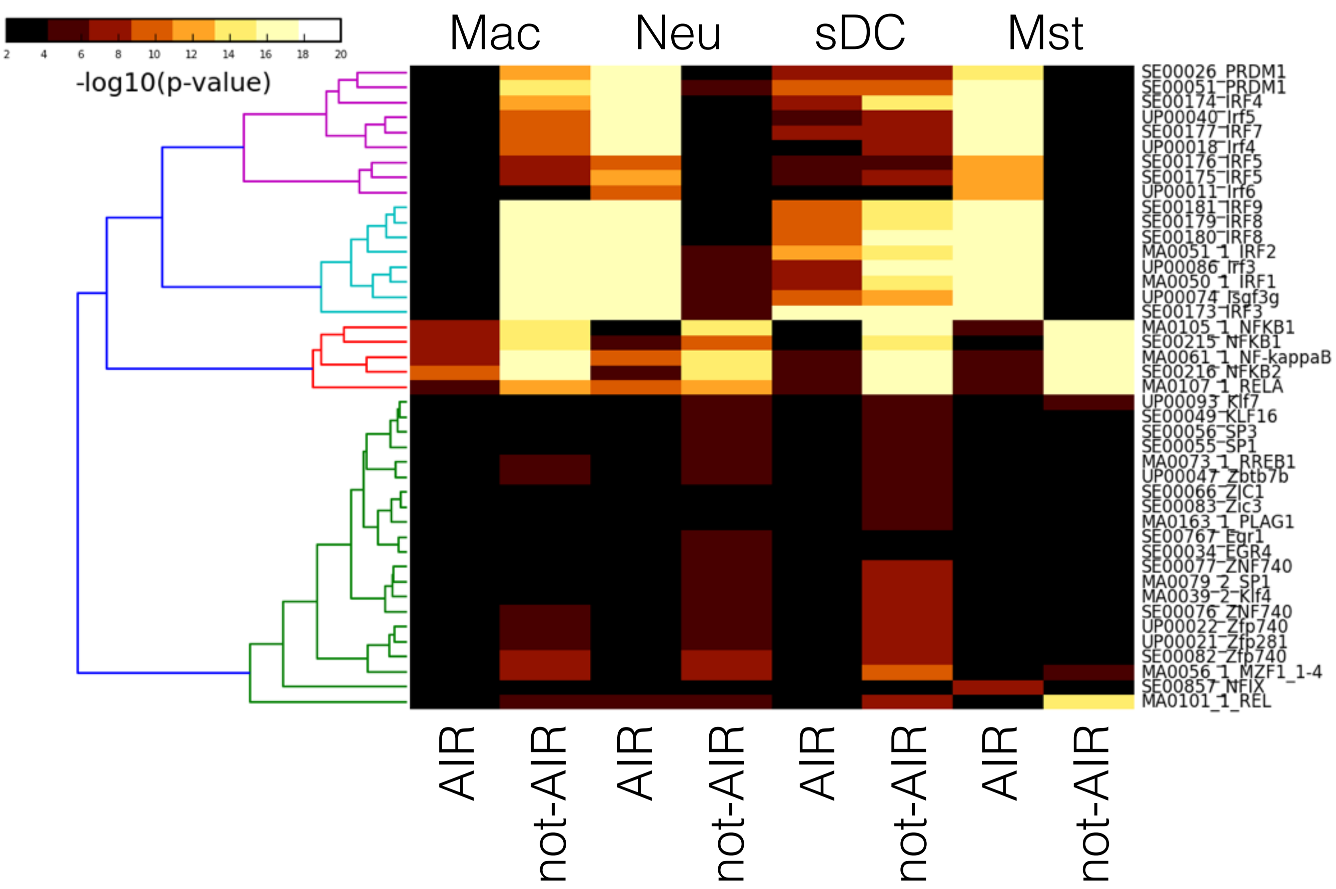

Figure S10. The full annotated version of Figure 5B. Transcription factor motif names are indicated on the right. The first two letters of the annotated name indicate the database origin; MA=JASPAR, UP=UniProt, SE=Jolma et al., 2013; Cell.

Figure S11

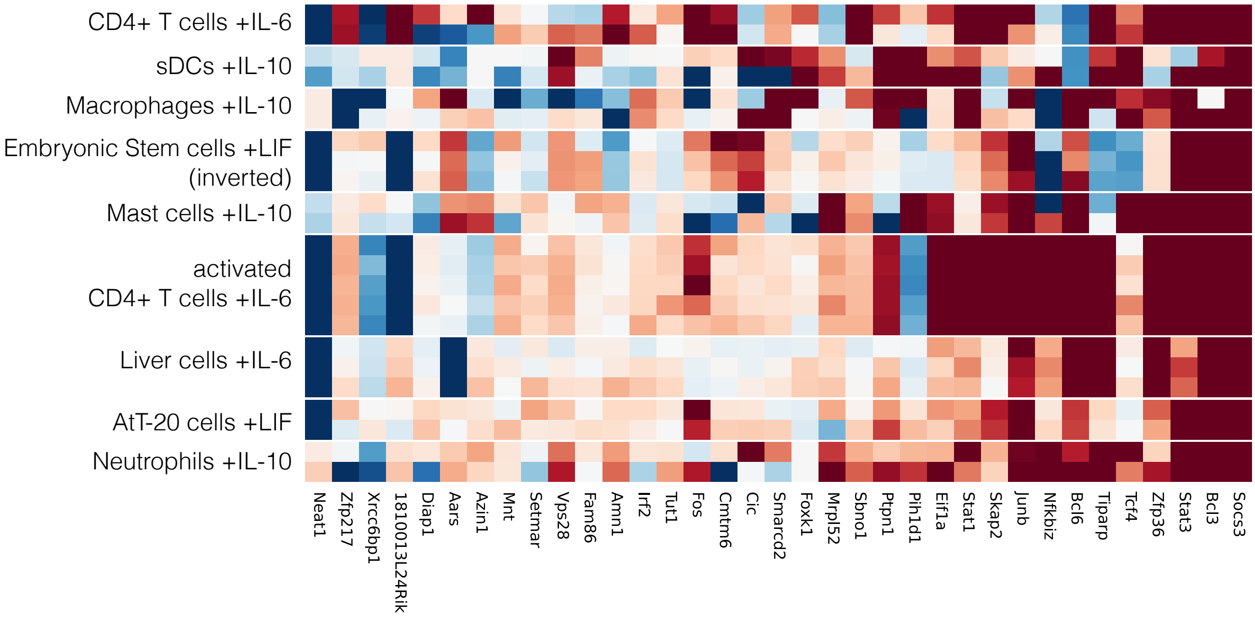

Figure S11. IL-10/STAT3 activates a 'core' of genes in a cell type independent manner. Updated heatmap from Hutchins et al., 2013; NAR, incorporating the new data from this study. The expression of the closest genes within 200 kb of the 'shared overlap' was measured in a series of gene expression microarray and RNA-seq data sets. Expression data are reversed in ESCs for clarity, but is otherwise down-regulated upon removal of LIF whereas all other treatments show up-regulation in response to cytokine stimulation. Data are from a CD4+ T cells treated with IL-21 (GSE19198), naive CD4+ T cells treated with IL-6 (GSE21671), ESCs upon withdrawal of LIF from the medium (GSE27708), peritoneal macrophages stimulated with IL-10 (GSE31529), AtT-20 cells treated with LIF (GSE19042), liver cells stimulated with IL-6 (GSE21060) and the RNA-seq samples described in this study (GSE55385).
